# Methods to extract and study the biological effects of murine gut microbiota using *Caenorhabditis elegans* as a screening host

**DOI:** 10.1371/journal.pone.0281887

**Published:** 2023-02-23

**Authors:** Claudia Miriam Alonzo-De la Rosa, Stéphanie Miard, Stefan Taubert, Frédéric Picard

**Affiliations:** 1 Institut Universitaire de Cardiologie et de Pneumologie de Québec, Québec, Canada; 2 Faculty of Pharmacy, Université Laval, Quebec, Canada; 3 British Columbia Children’s Hospital Research Institute, Vancouver, Canada; 4 Centre for Molecular Medicine and Therapeutics, The University of British Columbia, Vancouver, Canada; 5 Department of Medical Genetics, University of British Columbia, Vancouver, Canada; University of the Pacific, UNITED STATES

## Abstract

Gut microbiota has been established as a main regulator of health. However, how changes in gut microbiota are directly associated with physiological and cellular alterations has been difficult to tackle on a large-scale basis, notably because of the cost and labor-extensive resources required for rigorous experiments in mammals. In the present study, we used the nematode *Caenorhabditis elegans* as a model organism to elucidate microbiota-host interactions. We developed a method to extract gut microbiota (MCB) from murine feces, and tested its potential as food source for and its impact on *C*. *elegans* biology compared to the standard bacterial diet *Escherichia coli* OP50. Although less preferred than OP50, MCB was not avoided but had a lower energy density (triglycerides and glucose). Consistently, MCB-fed worms exhibited smaller body length and size, lower fertility, and lower fat content than OP50-fed worms, but had a longer mean lifespan, which resembles the effects of calorie restriction in mammals. However, these outcomes were altered when bacteria were inactivated, suggesting an important role of symbiosis of MCB beyond nutrient source. Taken together, our findings support the effectiveness of gut MCB processing to test its effects in *C*. *elegans*. More work comparing MCB of differently treated mice or humans is required to further validate relevance to mammals before large-scale screening assays.

## Introduction

Gut microbiota, defined as the community of microorganisms living in the intestinal tract, regulates numerous processes in the host such as innate immunity and energy metabolism [1–3]. For example, gut microbiota modulates lipid metabolism through the fermentation of non-digestible carbohydrates, triggering the production of short-chain fatty acids, which play a key role in the regulation of gut immune system [4]. Gut microbiota also influences behaviors through an axis linking peripheral intestinal functions and cognitive centers of the brain [5], notably due to its capacity to synthesize some neuroactive molecules and neurotransmitters [6]. To reverse dysbiosis, a disruptive imbalance in gut microbiota that can be detrimental for host health [7], microbiota can be transferred from the feces of a “healthy” donor into the gastrointestinal tract of a recipient [8,9]. In recent years, fecal microbiota transplantation has been used to study the specific effects of certain types of gut microbiota. However, despite the arduous research, many modulatory effects of the gut microbiota on host physiology are still elusive. In addition, considering the expensive and resource-consuming nature of these studies as well as the infinite possibilities associated with testing the differential impacts of microbiota modifications (for example, those induced by nutrition, environment, host age and genetics, pharmacological drugs, disease state, surgical interventions), more rapid and inexpensive screening tools are required before tackling these questions in mammals.

In recent years, *Caenorhabditis elegans* has emerged as a powerful tool to understand the complexity of microbiota-host interactions [10–13]. *C*. *elegans* is a transparent nematode worm used as a model organism to study all aspects of animal development, metabolism, behavior, and lifespan due to functional and genetic similarities with more complex organisms, including mammals [14]. As in humans and mice [15,16], the native microbiome of *C*. *elegans* is composed mainly by the phyla of bacteria *Bacteroidetes*, *Actinobacteria*, *Firmicutes*, and *Proteobacteria* [17]. Most interestingly, this nematode feeds on bacteria, which allows its microbiota to be easily modified and studied [13]. Moreover, as in mammals, bacteria can establish a symbiotic relationship with *C*. *elegans* [13,18]. Consistent with this, *C*. *elegans* has been widely used to study other specific *E*. *coli* strains and numerous other bacteria and fungi that are part of the human gut microbiota, both commensals (e.g., *Lactobacillus gasseri*, *L*. *delbrueckii*, *L*. *fermentum*, *Bifidocaterium animalis lactis*) and pathogens (e.g., *Enterococcus faecalis*) [13,19]. Changes in maximal longevity, reproduction, locomotion, and behaviors have been reported, in part associated with the different nutritional value of these specific strains isolated in controlled laboratory environments [10,20–30]. Based on these studies and considering the resources necessary to study the effects gut microbiota in mammals, we hypothesized that *C*. *elegans* could serve as a valid model to rapidly screen for traits induced by microbiota extracted from feces. To address this, we have developed a protocol to extract and characterize the gut microbiota (MCB) harvested from murine feces and to assess its influence on the physiology and healthspan of *C*. *elegans*.

## Materials and methods

### *C*. *elegans* and *E*. *coli* cultures

Wild-type *C*. *elegans* N2 and *E*. *coli* OP50 strains were provided by the *Caenorhabditis* Genetics Center (CGC). *E*. *coli* OP50 was grown overnight in liquid Terrific Broth (TB) at 37°C with agitation [31]. Worms were maintained and grown at 20°C on solid nematode growth media (NGM) plates seeded with *E*. *coli* OP50 cultures [32]. Worms were synchronized with the alkaline hypochlorite method as described [32].

### Feces harvest

Male C57BL/6J mice of 3 months of age were purchased from Jackson Labs (#000664). Mice had *ad libitum* access to regular chow diet and water and were housed individually under a 12:12 hrs dark:light cycle at an ambient temperature of 23 ± 1°C. Mice were cared for and handled in accordance with the Canadian Guide for the Care and Use of Laboratory Animal and the Université Laval Institutional Animal Care Committee approved the protocols.

On the morning of harvest, approximately 3 hours after lights were switched on, mice were placed in groups of 3 in cages containing no bedding. Two hours later, feces that were not in contact with urine were taken with ethanol-disinfected, small surgical pliers and placed in tubes. Feces were then kept at room temperature and processed on the same day for microbiota extraction.

### Microbiota extraction

To extract gut microbiota (MCB), feces were put in either PBS buffer or M9 (1 mL per 100 mg of feces) and gently homogenized with agitation for 3 min. Since 40–60% of the bacteria residing within the gut are individually unculturable, this method represents an advantage when culturing the gut microbiota because this avoids changes in bacterial composition [33,34] by selectively gaining or losing strains. The mixture was then centrifuged at 800 *g* for 3 min and the supernatant was filtered on a cell strainer with a pore size of 70 μm to eliminate any insoluble material.

### Bacterial DNA extraction and quantification

This protocol was adapted from [35]. Briefly, 455 μL of EtNa DNA extraction reagent (240 Mm NaOH, 74% ethanol, 2.7 mM EDTA) was mixed with 100 μL of bacterial solution (either from MCB homogenates or cultured *E*. *coli* OP50) and heated at 80°C for 10 minutes before DNA extraction using QIAmp mini spin column (QIAGEN) following manufacturer’s instructions. Quantification of extracted bacterial DNA was performed on a BioDrop analyser (Montreal Biotechnologies Inc., Canada). This method allowed the distribution of similar bacterial quantities between groups for proper comparison. Pilot experiments were performed testing the necessary quantity of bacteria to avoid food restriction by the observation of bacterial lawns left after a given period (for a same starting amount of bacterial DNA and worms per plate) (S1 Table in S1 File).

### Bacterial viability

This protocol was adapted from [34]. Aliquots containing 5 μg of bacterial DNA were pelleted and resuspended in 1 mL of PBS or M9, of which 90 μL were mixed with 10 μL of PBS or M9 containing resazurin (0.125 mg/mL) in 96-well plates. Plates were then incubated for 1 h at room temperature, and the resulting fluorescence was quantified (530 nm excitation and 590 nm emission) in a hybrid multi-mode reader (BioTek).

### Schaeffer-Fulton endospore staining

One droplet of the MCB was heated, fixed on a glass slide, covered with a square of paper towel, saturated with a malachite green stain solution (0.5% w/v in water), and steamed for 5 min over a glass container of boiling water to keep constant moisture. After removal of the paper, samples were washed with distilled water. Subsequently, a safranin stain solution (10 mL of 2.5% w/v in ethanol 95% and 90 mL of distilled water) was added and after 30 seconds, the samples were washed again with distilled water. Images were taken on an inverted microscope. With this staining method, bacterial endospores appear bright green and vegetative cells are stained red.

### Nutritional content

Based on DNA content, similar quantities (30 μg DNA) of MCB homogenates and *E*. *coli* OP50 cultures were pelleted and either 200 μL of distilled water (for glucose and glycogen measurements) or 500 μL of NP40 5% solution (for glycerol analysis) were added to samples. Samples were subjected to 10 cycles of sonication (Branson Sonifier Cell Disruptor 185; LabX, USA) with an amplitude of 50%. Each cycle consisted of 30 sec on and 30 sec off. Between each cycle, samples were put on ice to avoid overheating. Metabolites were quantified by colorimetry using kits # K646 for glycogen and glucose and # K622 for glycerol (Bio Vision, USA).

Triglycerides were extracted by the Folch method as described [36], using 30 μg of bacteria resuspended in 50 μL PBS. Dried samples were resuspended in 60 μL of isopropanol and triglycerides were quantified by colorimetry (Randox, UK) according to the manufacturer’s instructions.

### Bacterial inactivation

MCB homogenates and *E*. *coli* OP50 cultures were either inactivated by heating (30 min at 65°C) or with paraformaldehyde (PFA) using a protocol adapted from [37]. Briefly, PFA was added to 50 mL tubes containing MCB homogenates or *E*. *coli* OP50 cultures to obtain final concentrations of 0.5%. Tubes were then agitated at room temperature, 200 rpm for 1 h. Subsequently, tubes were centrifuged 3000 *g* for 20 min, and pellets were washed 5 times with 25 mL PBS to remove all trace of PFA.

### Pharyngeal pumping

Synchronized L4 worms were singled onto NGM peptone-free 24-well plates with MCB or *E*. *coli* OP50 (each containing 5 ng DNA). Pharyngeal pumping was counted in triplicate in individual worms for 1 min at days 1, 2, and 5 using an Olympus MVX10 microscope. Pharyngeal pumping was determined as the average of the count by two independent observers on the same worms.

### Food choice assays

MCB and *E*. *coli* OP50 (each containing 5 μg DNA) were seeded either in the middle (in one-food choice assays) or side by side (1.5 cm from the center of the plate) on standard 30 mm NGM peptone-free plates containing 50 μM 5-Fluoro-2′-deoxyuridine (FUDR). Synchronized L4 worms were placed on food and located 1, 24, 72 and 120 h later under microscope. Worms outside of any bacterial lawn were counted as not on food. The proportion of worms on each food was calculated and graphed.

### Lifespan assays

Synchronized L4 worms were seeded on NGM peptone-free plates with FUDR 50 μM and MCB or *E*. *coli* OP50 (each containing 10 μg DNA) and kept at 20°C. Worms were transferred to a new plate every two days until death. Worms were considered dead when they failed to respond to prodding. Animals that crawled off the plates, killed by the investigator, died of drying on the edges of the plate, bagging, or vulva bursting were excluded from lifespan assays.

### Quantifying bag of worms (Bag) and age-associated vulval integrity defect (Avid) phenotypes

Synchronized L4 worms were seeded on NGM peptone-free plates seeded with either MCB or *E*. *coli* OP50 (each containing 10 μg DNA) and kept at 20°C. Worms were transferred to a new plate every day until the end of the reproductive period. After this period, no more Bag and Avid phenotypes were observed. The proportion of Bag and Avid worms were calculated and graphed.

### Brood size

Approximately 30 synchronized L4 worms were singled onto NGM peptone-free 24-well plates with MCB or *E*. *coli* OP50 (each containing 5 ng DNA). Worms were transferred to a new well daily until they stopped laying eggs, and the number of eggs was counted every day. Brood size was determined as the sum of total eggs laid by each individual worm.

### Nile red staining

Approximately 200 synchronized L4 stage worms were placed on NGM peptone-free plates with 50 μM FUDR and *E*. *coli* OP50 or MCB (each containing 100 μg DNA distributed in 10 droplets). Nile red staining was performed 3, 9, and 15 days later [31]. Briefly, worms were washed twice with 0.01% Triton-x-100 in M9 buffer, resuspended in 100 μL of 40% isopropanol, and incubated 3 min at room temperature. Worms were then washed twice with 1 mL of M9 buffer. Subsequently, a Nile red staining solution (6 μL of Nile red stock solution (0.5 mg/mL in acetone) added to 1 mL of isopropanol 40%), was added to each sample and worms were incubated for 2 h at room temperature with agitation and protected from light. After washing with M9 buffer, nematodes were incubated with 600 μL of 0.01% Triton in M9 for 30 min at room temperature, washed, and resuspended in M9 buffer. Images were taken on an epifluorescence microscope (EVOS life technologies, USA). Nile red fluorescence intensity was measured with a Biosorter flow cytometer (Union Biometrica, MA, USA) and normalized to individual worm size (as indexed by time of flight, TOF) as described [38].

### Oil red O staining

Approximately 20 synchronized L4 worms were placed on NGM peptone-free plates with 50 μM FUDR and *E*. *coli* OP50 or MCB for. Oil red O staining was performed 3, 9, and 15 days later as described [39]. Briefly, a 0.5% Oil red O stock solution in high-quality 100% isopropanol was prepared, left overnight, and filtered through a 0.45 μm filter. The day before use, the Oil red O stock solution was diluted to 60% with double distilled water, filtered through a 0.45 μm filter, left overnight, and filtered again through a 0.45 μm filter before use. Simultaneously, worms were washed two times with M9 buffer and resuspended in 60% isopropanol. Subsequently, the 60% Oil red O filtered solution was added to each sample and worms were incubated overnight at room temperature. After washing with M9 buffer, the nematodes were resuspended in Triton 0.01% in M9. Images were taken by an Olympus MVX10 microscope.

### Statistical analysis

All data are expressed as means ± SEM. Bacterial viability was analyzed by ANOVA repeated measures. Student’s t-tests were used to analyze nutritional value of MCB homogenates and *E*. *coli* OP50 cultures. Two-way ANOVA was used to analyze food choices, body size, brood size, Bag phenotype, Avid phenotype, relative fat mass, and pharyngeal pumping. Nematode survival was analyzed by Kaplan–Meier method and survival differences were tested for significance using the log-rank test against controls animals by the OASIS application [40].

## Results and discussion

### Viability of MCB

**Fig 1:**
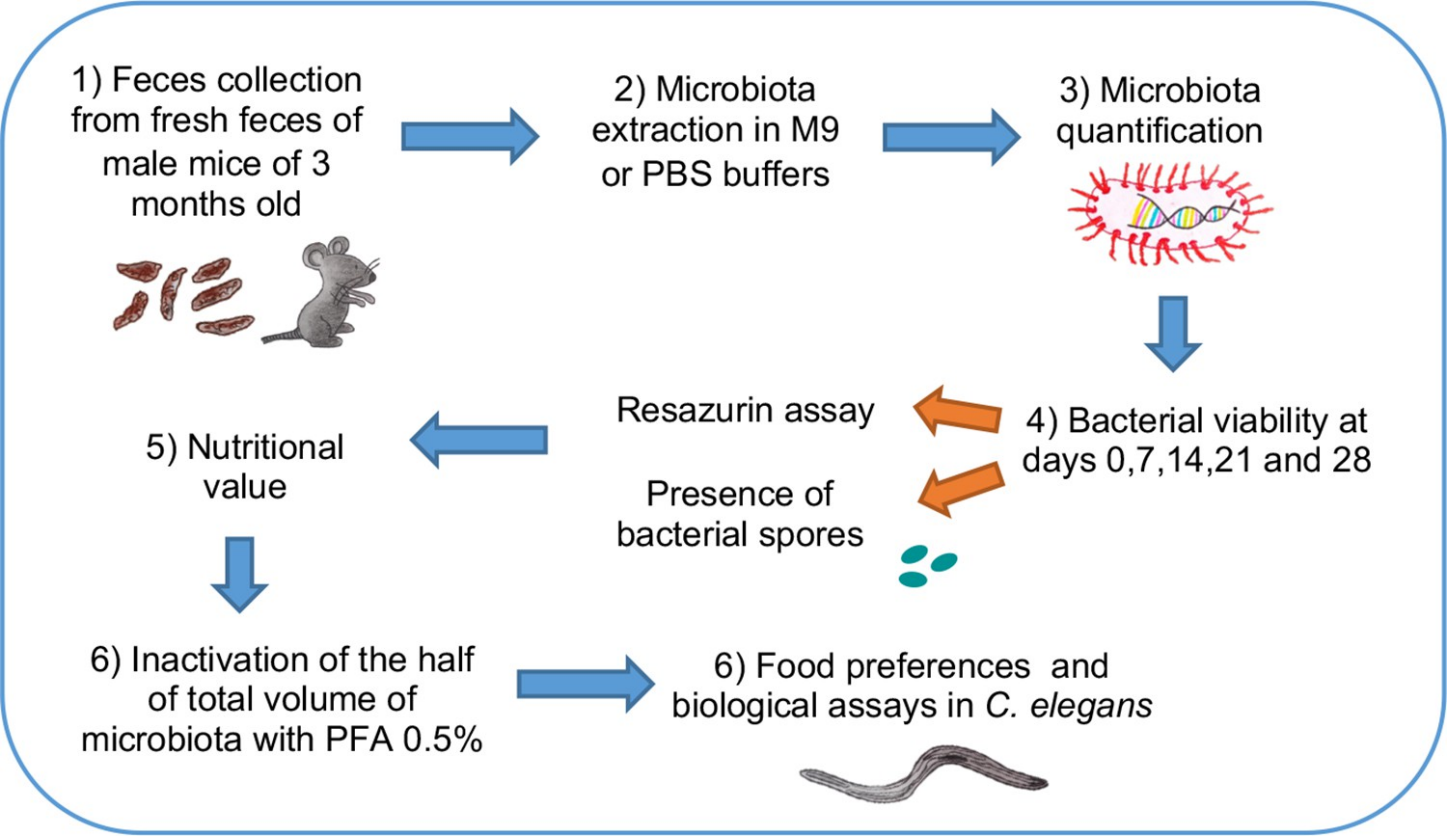
Outline of experimental design. Fresh feces are collected from mice. MCB homogenates are produced by immersing the feces in M9 or PBS buffers, and quantified by bacterial DNA analysis. Bacterial viability is then evaluated quantitatively by resazurin assay (Alamar blue) and qualitatively by the presence of spores (Schaeffer-Fulton staining). Nutritional value of the MCB is evaluated by quantifying energy metabolites (glucose, glycogen, triglycerides, and glycerol). Subsequently, half of MCB extract is inactivated with PFA and the other half is kept alive to allow symbiosis. Alive and inactivated MCB homogenates are then used to characterize phenotypes and biological traits in the nematode *C*. *elegans*.

**Fig 2:**
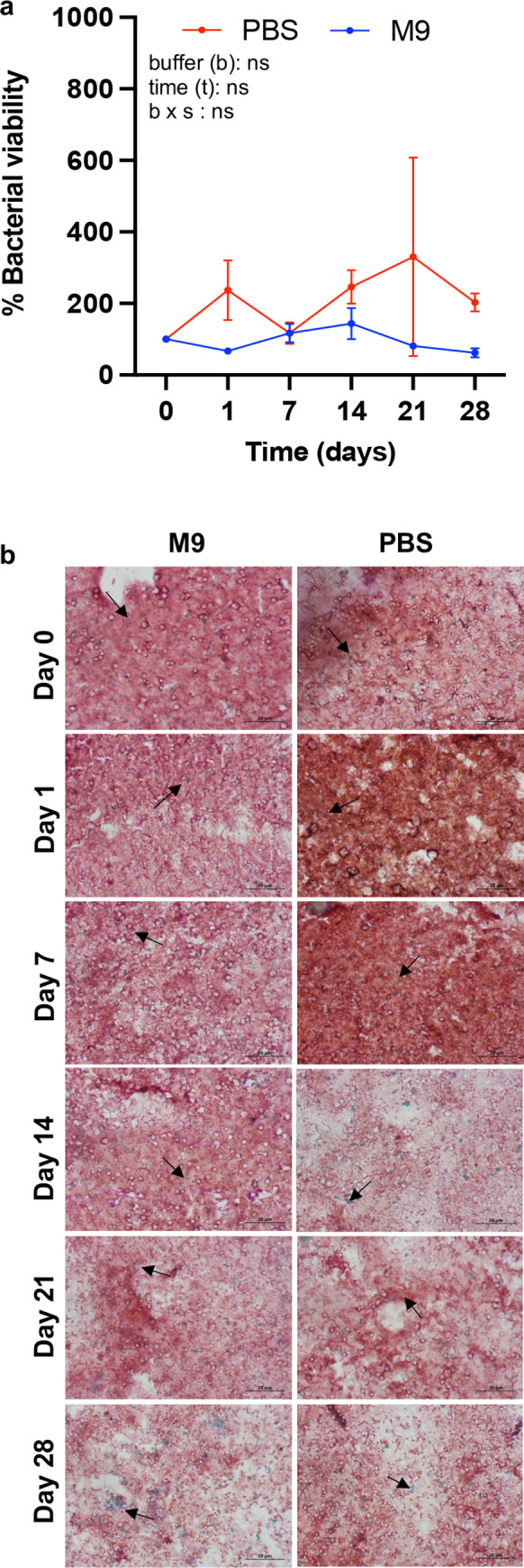
Bacterial viability of fecal MCB homogenates. **(a)** Quantitative evaluation of bacterial viability in MCB homogenates obtained after immersion of fresh feces of male mice in either M9 or PBS over 28 days. Bacterial viability at day 0 (after feces collection and homogenization) is considered as 100%. Mean ± SEM values. Each data point represents the average of three resazurin assays in technical duplicates. Statistical comparisons by ANOVA repeated measures. (**b)** Representative images of Schaeffer-Fulton staining (100x) at days 0, 1, 7, 14, 21 and 28. This staining allows the qualitative evaluation of bacterial viability by coloring spores, which are an indicator of viability. Spores are green and bacterial vegetative cells are red—pink. Spores are indicated by an arrow on each image.

A general workflow of MCB processing is presented in Fig 1. Because sample storage buffer can influence the viability of bacteria over time [41,42], we first used a resazurin assay to evaluate viability in MCB homogenates kept in either PBS or M9 buffers at 1-week intervals for 4 weeks. No significant differences were observed between buffers over this period (Fig 2A). Supporting these results, Schaeffer-Fulton staining indicated the presence of spores produced in all samples over that period (Fig 2B). These spores were likely produced in response to adverse conditions (PBS and M9 containing no energy substrate), possibly from spore-producing *Firmicutes*, which at 56% is the dominant phyla in the gut microbiota of mice [43,44]. Thus, MCB remained viable independently of storage buffers, consistent with previous reports that PBS preserves Gram-positive and Gram-negative bacteria at room temperature for several years [42]. Based on these findings, additional experiments described below were performed using PBS as buffer to extract and maintain fecal MCB.

### Nutritional value and intake of MCB

**Fig 3:**
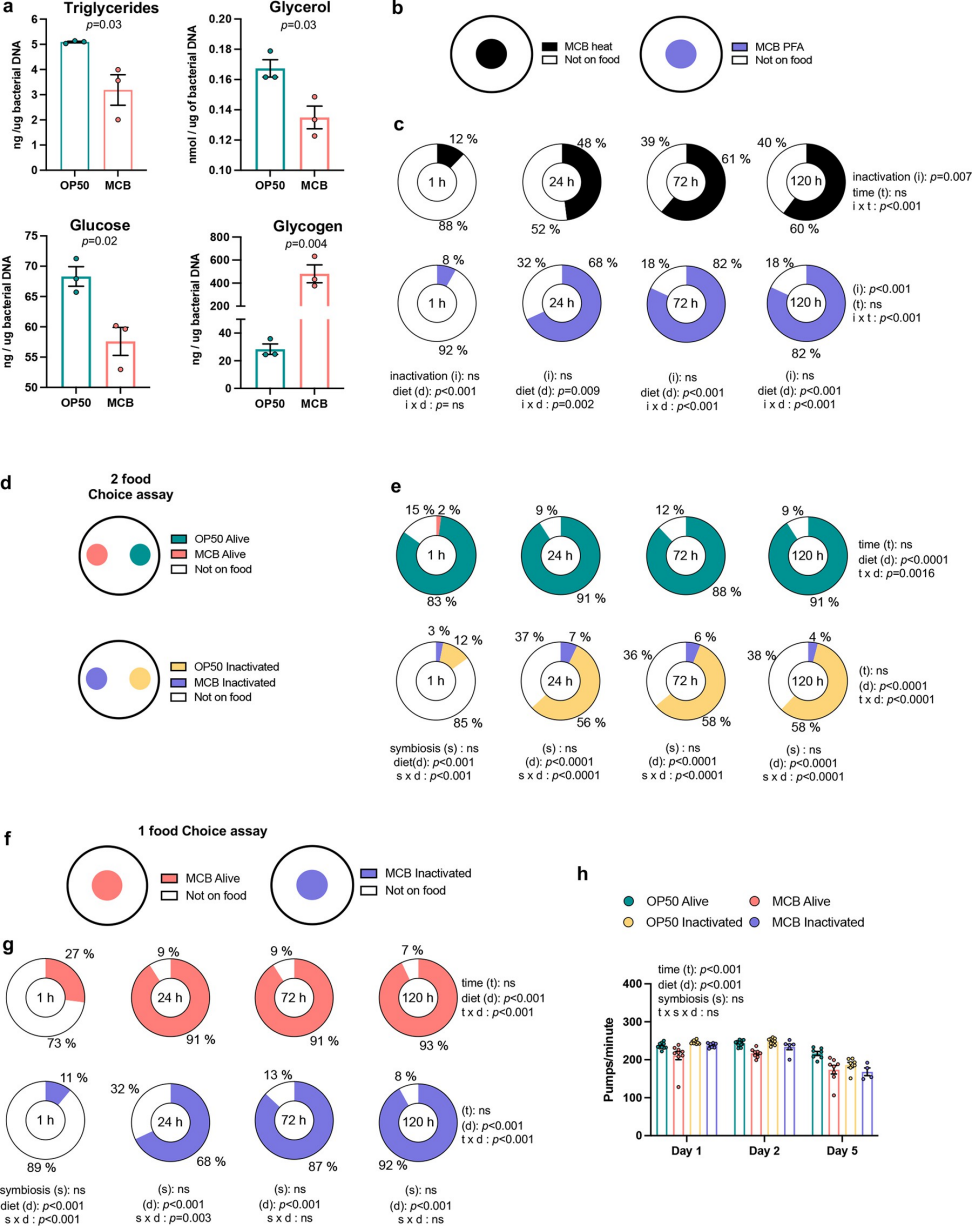
Bacterial food quality of MCB and *C*. *elegans* food intake and preferences. (**a)** Concentrations of triglycerides, glycerol, glucose and glycogen in *E*. *coli* OP50 and in MCB homogenates described in Fig 2, as determined based on similar DNA content. Each bar represents the average of three measurements performed in technical duplicates. **(b)** Schematic illustration of food preference assay between heat-inactivated versus PFA-inactivated microbiota. (**c)** Percentage of adult worms on and off the bacterial lawn. n = 40 worms/replicate for a total of 120 worms. (**d)** Schematic illustration of food choice assay between two different bacteria. (**e)** Percentage of adult worms on and off of the bacterial lawns. n = 40/replicate for a total of 240 worms. (**f)** Schematic choice assay between alive versus inactivated MCB. (**g)** Percentage of adult worms on and off the only one bacterial lawn. n = 40 /replicate for a total of 240 worms. (**h)** Pharyngeal pumping per minute of worms fed with *E*. *coli* OP50 or MCB either alive or inactivated. Statistical comparisons by 2 x 2 ANOVA. ns: Not significant.

As in mammals [45], bacterial diets modulate the phenotypes of *C*. *elegans* through their composition in macronutrients as well as symbiosis with their host [11,20,21,23,24]. We hypothesized that any differential outcome obtained after feeding worms with live bacteria compared to feeding with inactivated bacteria (thus most exclusively serving as nutrient source) would reveal effects that could be attributed to the impact of interactions with *C*. *elegans*. Interestingly, per same amount of bacterial DNA, we found that *E*. *coli* OP50 contained higher levels of triglycerides (*p* = 0.007), glycerol (*p* = 0.03), and glucose (*p* = 0.02) than MCB, but ten-fold lower glycogen levels (*p* = 0.004, Fig 3A).

Two published methods were next tested for bacterial inactivation, treatments with heat [46] and PFA [37]. Both equally and completely inhibited viability as assessed by resazurin assays (S2 and S3 Tables in S1 File). However, PFA permeabilizes bacterial cells, which preserves their overall structure and keeps them edible for worms [37]. In addition, a report suggested that heat-killed bacteria are a low-quality food that *C*. *elegans* cannot use [46]. Thus, these methods were evaluated for their possible interference towards food intake using a food aversion test by counting the number of worms on each bacterial lawn at 1, 24, 72, and 120 h (Fig 3B). Worms had significantly greater avoidance for heat-killed MCB than for PFA-treated MCB over time (Fig 3C, *p*<0.0001). Based on these findings, additional experiments described below were performed using PFA 0.5% to inactivate fecal MCB and *E*. *coli* OP50.

In addition to nutrient content, bacterial metabolism influences *C*. *elegans* food choices [37,47,48], which likely contributes to preferences for certain bacterial diets and avoidance of other pathogenic, detrimental strains [23,48,49], and triggers dwelling/roaming phases through sensory perception by amphid neurons [29,30]. These concepts were tested using two-food choice assays in which worms were given the choice between lawns of OP50 or MCB in same quantities, either live or inactivated (Fig 3D). Compared to inactivated bacteria, live bacteria preferentially attracted worms as early as after 1 h of food access (Fig 3E, *p*<0.0001). This difference remained throughout the experiment, although it tended to diminish with time (Fig 3E). Worms also clearly preferred OP50 over MCB either alive (*p*<0.0001) or inactivated (*p*<0.0001), with only 2–7% of worms choosing MCB in these conditions over the course of the experiment (Fig 3E). Finally, to determine whether worms were more attracted to OP50 or rather avoided MCB, we performed a one-food choice assay where MCB (either alive or inactivated) was the only food option (Fig 3F). This assay revealed that the MCB did not induce an avoidance response, as the majority of worms dwelled on both live and inactivated 24 h after being in the presence of MCB (Fig 3G). Because worms were cultured on OP50 from hatching to L4 before being in contact with either OP50 or MCB, it is also important to note that habituation and/or epigenetic drivers towards OP50 contributed to diet preference.

Rates of pharyngeal pumping were next measured as a complement to the food choice assay because they directly regulate ingestion of bacteria [50]. This behavior is regulated by the extrapharyngeal nervous system, humoral factors, internal nutrition status, and external cues [51]. Worms fed with MCB showed decreased pharyngeal pumping per minute compared to those fed on OP50 when tested 1, 2, and 5 days after being in contact with the diets (Fig 3H, *p*<0.0001). The exact neuronal circuits involved in these behaviors remain unknown. Taken together, these results strongly suggest that, although not as preferred or high energy-dense as OP50, murine fecal MCB unlikely sends pathogenic cues to *C*. *elegans*, and that worms can readily feed on gut MCB obtained with the above-mentioned method for harvesting and processing feces.

### Impact of MCB on *C*. *elegans* development

**Fig 4:**
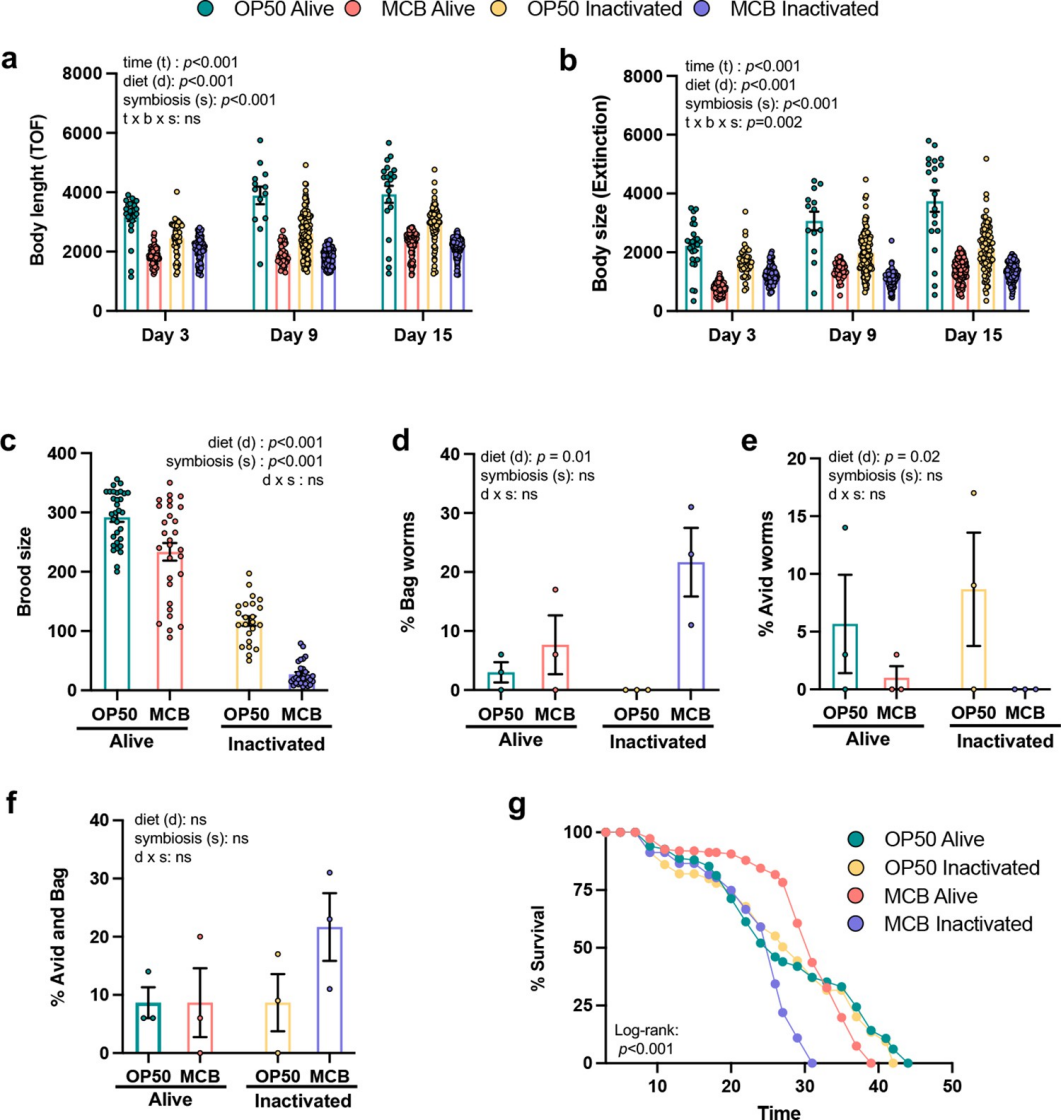
Comparative effects of MCB on reproduction and healthspan of *C*. *elegans*. (**a)** Body length based on relative axial length (Time of flight, TOF) of worms fed *E*. *coli* OP50 versus MCB either alive or inactivated for the indicated time points. Each point represents an individual worm. **(b)** Body size of worms fed *E*. *coli* OP50 versus MCB either alive or inactivated. Each point represents an individual worm. (**c)** Brood size of worms fed *E*. *coli* OP50 versus MCB either alive or inactivated. 30 worms were tested individually per group. **(d)** Percentage of worms showing a Bag phenotype over the reproductive period in worms fed with *E*. *coli* OP50 or MCB either alive or inactivated. Data represent the mean ± SEM values on 35 worms/replicate for a total of 105 worms. **(e)** Percentage of worms showing an Avid phenotype over the reproductive period in worms fed with *E*. *coli* OP50 or MCB either alive or inactivated. Data represent the mean ± SEM values on 35 worms/replicate for a total of 105 worms. Statistical comparisons by 2 x 2 ANOVA. ns, no significant. (**g)** Survival curves of worms fed on *E*. *coli* OP50 versus MCB either alive or inactivated. n = 150 per group, see also S4 Table in S1 File).

Fecal microbiota transplantation experiments have indicated that gut microbiota modulates metabolism and healthspan in mammals [52,53]. Individual bacterial diets differentially modulate the development and phenotypic traits of *C*. *elegans* as well [21,23,47]. Based on this concept, we aimed at confirming that worms were not only able to feed on gut MCB but use it for normal development and reproduction. To this end, we first took advantage of the rapid sorting analyzer for pluricellular organisms (Biosorter) and analyzed worm length and size by probing for time-of-flight (TOF) and extinction, respectively [37,38]. In agreement with previous reports [37,46], worms fed with inactivated bacteria were smaller in length (Fig 4A *p*<0.001) and size (Fig 4B *p*<0.001) compared to those fed with live bacteria, especially after 9 days on these diets. Worms fed with MCB also showed reduced body length (Fig 4A, *p*<0.001) and size (Fig 4B, *p*<0.001) compared to those fed with *E*. *coli* OP50. Consistent with these findings, brood size was ~20% lower in worms fed MCB in comparison with those fed OP50 (Fig 4C, *p*<0.001). Interestingly, MCB-fed groups showed a higher rate of Bag (Fig 4D, *p =* 0.01, defined as the retention and hatching of eggs inside the parental body) but lower frequency of age-associated vulval integrity (Avid; Fig 4E, *p =* 0.02) phenotypes than those fed with OP50. When combined, these traits were no longer different between MCB and OP50-fed groups (Fig 4F). Inactivation of bacterial diets further reduced brood size (Fig 4C *p*<0.001), but it did not have a significant effect on the percentage of Bag or Avid worms (Fig 4D–4F). These results suggest that, whereas worms can feed normally on gut MCB, this diet is less optimal for development and reproduction than OP50. More work is required to uncover the neural circuits involved in these egg-laying behaviors and developmental defects [54].

Since changes in brood size and Bag/Avid phenotypes are associated with variations in longevity [55,56], lifespan curves were analyzed and compared between groups. Surprisingly, although inactivation of OP50 triggered reduced body length and brood size, it had no impact on mean or maximal longevity (Fig 4G *p*<0.001, S4 Table in S1 File). However, worms fed with live MCB exhibited a higher mean lifespan than those fed with OP50, whereas those fed with inactivated MCB were characterized by a reduced lifespan, both mean and maximal (Fig 4G). Interestingly, these changes did not parallel those in Avid and Bag phenotypes, both of which associated with early death [55,56]. More work is required to unravel the mechanisms responsible for the observed effects of MCB on longevity, notably considering the likely important contribution of symbiosis.

**Fig 5:**
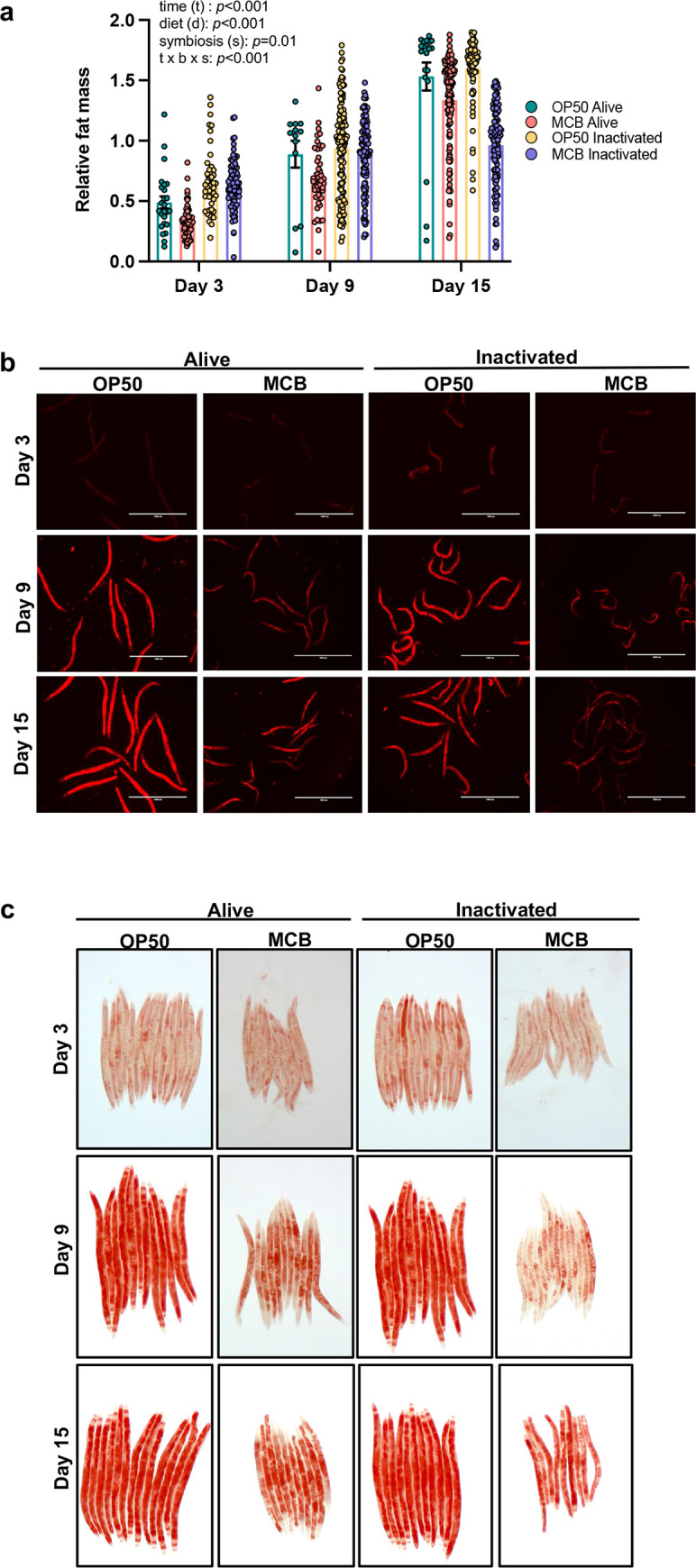
Effects of OP50 and MCB on fat accumulation. **(a)** Nile red fluorescence relative to length (TOF) in worms fed *E*. *coli* OP50 or MCB either alive or inactivated for up to 15 days. Each point represents an individual worm. Statistical comparisons by 2 x 2 ANOVA followed by Bonferroni’s multiple comparison test. ns, no significant. (**b)** Representative images of Nile red staining at days 3, 9 and 15 days in worms described in (a) Scale bar: 1000 μm. **(c)** Representative images of Oil red O staining at days 3, 9 and 15 in worms described in (a). Magnification 25x.

In mammals, gut microbiota is an important regulating factor of fat storage [57]. Similarly, in *C*. *elegans*, fat content is greatly variable depending on the bacterial diet [23] and is modulated by development and fertility. Thus, we characterized the impact of fecal MCB on fat stores using fluorescent Nile red staining and Oil red O in fixed worms. Nile red fluorescence was rapidly quantified by Biosorter and normalized to TOF to account for body length. MCB robustly diminished fat stores of *C*. *elegans* relative to OP50 (p<0.001, Fig 5A). Inactivation of bacteria by PFA also reduced relative fat mass in worms either fed *E*. *coli* OP50 or MCB (*p* = 0.01, Fig 5A). These findings were supported by microscopy (Fig 5B). Oil red O staining further corroborated the robust effect of MCB on fat accumulation (Fig 5C), without impacting fat distribution, mainly observed in the intestine, hypodermis, and gonad (Fig 5C). These findings are consistent with the lower energy density of MCB compared to that of OP50 (Fig 2A).

Previous studies suggested that *C*. *elegans*, through amphid neurons, makes dietary choices basing on the quality of food and its impact on survival and reproduction [29,58]. The findings of the present study are consistent with this concept. Worms had much less preference for MCB compared to *E*. *coli* OP50. Reduced body length, brood size, and fat mass observed in MCB-fed worms were associated with the relatively less energy-dense characteristic of MCB compared to OP50 (especially triglycerides). In turn, the MCB diet had a positive impact on mean lifespan, possibly due to a calorie-restriction effect, especially from glucose [59]. These characteristics were strongly modified by bacterial inactivation, convincingly adding to recent evidence that bacterial metabolism and interactions with the host is possibly as important as bacterial composition, at least for some species [37]. More studies are required to elucidate the potential mechanisms of this phenomena.

## Conclusions

We report herein a simple method to extract and use gut microbiota to test for biological effects in the model organism *C*. *elegans*. Although not as preferred and energy-dense as *E*. *coli* OP50, fecal MCB is a suitable, unlikely pathogenic food source that supported *C*. *elegans* development, reproduction, and lifespan, especially when kept alive. How symbiosis improves host biology in *C*. *elegans* remains to be elucidated. Since *C*. *elegans* shares many features with mammals, including genetic regulatory pathways [13,60], the present protocol could allow shorter experimentation times and much lower costs before embarking on more complicated studies in mice or humans, in particular during initial screenings. However, more phenotypic comparisons following feeding with MCB of mice or humans with different treatments/conditions are required to further validate relevance to mammalian traits before large-scale assays.

## Supporting information

S1 File This file contains supplementary tables containing data. (DOCX)

S1 Data This file contains the minimal data set underlying the results. (XLSX)
