# Supplementary material for: Methods to extract and study the biological effects of murine gut microbiota using Caenorhabditis elegans as a screening host
Source: PLoS One. 2023 Feb 23;18(2):e0281887. doi: 10.1371/journal.pone.0281887 (PMC9949637; doi:10.1371/journal.pone.0281887)
Supplement: S1 File — (DOCX) [file pone.0281887.s001.docx]

**SUPPLEMENTARY TABLES**

**S1 Table. Quantity food validation.** Validation of quantity of food for *C. elegans* cultures to avoid caloric restriction over 43 hours. The number of + is directly proportional to the amount of food left on the plates.

| # Exp | 1 | | 2 | | 3 | |
| --- | --- | --- | --- | --- | --- | --- |
| **Group** | **OP50** | **MCB** | **OP50** | **MCB** | **OP50** | **MCB** |
| **0 h** | | | | | | |
| **10 μg** | ++++ | ++++ | ++++ | ++++ | ++++ | ++++ |
| **20 μg** | ++++ | ++++ | ++++ | ++++ | ++++ | ++++ |
| **30 μg** | ++++ | ++++ | ++++ | ++++ | ++++ | ++++ |
| **27 h** | | | | | | |
| **10 μg** | ++ | ++ | ++ | ++ | ++ | ++ |
| **20 μg** | +++ | +++ | +++ | +++ | +++ | +++ |
| **30 μg** | ++++ | ++++ | ++++ | ++++ | ++++ | ++++ |
| **43 h** | | | | | | |
| **10 μg** | ++ | + | ++ | + | ++ | + |
| **20 μg** | +++ | ++ | +++ | ++ | +++ | ++ |
| **30 μg** | +++ | +++ | +++ | +++ | +++ | +++ |

**S2 Table. Bacterial viability of MCB**. Bacterial viability was measured by direct by direct microplate resazurin assay immediately after PFA or Heat treatments. Bacterial viability of alive MCB was considered as 100%.

| Inactivation | % Bacterial viability |
| --- | --- |
| No inactivation | 100% |
| PFA 0.5 % | 0 |
| Heat killed | 0 |

**S3 Table. Viability of PFA-treated MCB.** Bacterial viability was measured by direct by direct microplate resazurin assay at different time.

| PFA inactivation | |
| --- | --- |
| Time after inactivation | **% Bacterial viability** |
| 1h | 0 |
| 1 day | 0 |
| 7 days | 0 |
| 14 days | 0 |
| 21 days | 0 |
| 28 days | 0 |

**S4 Table.** Survival of N2 worms on OP50 or MCB either alive or inactivated starting at L4 worm stage.

| Group | n | Median lifespan  (days) | Mean lifespan (days) ± SEM | Bonferroni *p* value |
| --- | --- | --- | --- | --- |
| OP50 | 150 | 26 | 27.7 ± 0.8 | 0.9817 |
| MCB | 150 | 31 | 30.1 ± 0.6 |  |
| OP50 Inactivated | 150 | 29 | 27.4 ± 0.9 | <0.001 |
| MCB Inactivated | 150 | 26 | 23.5 ± 0.5 |  |
